# Investigating into the dual role of loan loss reserves in banking production process

**DOI:** 10.1007/s10479-021-04365-w

**Published:** 2021-12-01

**Authors:** Hirofumi Fukuyama, Yong Tan

**Affiliations:** 1 Faculty of Commerce, Fukuoka University, 8-19-1 Nanakuma, Jonan-Ku, Fukuoka, 814-0180 Japan; 2 Department of Accounting, Finance and Economics, Huddersfield Business School, University of Huddersfield, Huddersfield, HD1 3DH Queensgate UK

**Keywords:** Network DEA, Costly disposability, Banking

## Abstract

This paper considers the use of loan loss reserves (LLRs) in the banking production process and treats it as one variable with a dual role. We establish a three-stage network Data Envelopment Analysis model to address this issue. Using a sample of 43 Chinese commercial banks over the period 2011–2019, the results show that the banks with the ratio between LLRs and total loans less than 1% have higher level of efficiency compared to the ones holding the ratio greater than 1%. The results show that when excluding LLRs in the production process, the efficiency scores are significantly inflated. We find that small and medium sized banks are more efficient than their big counterparts, however, the results show that big banks hold more than enough amounts of LLRs than the one required by the regulatory authority. When LLRs are excluded from the production process, it shows that big banks perform better than small and medium sized banks. Our findings show that less liquid banks perform better than the ones with higher levels of liquidity no matter in which way LLRs are treated. Finally, we find that lower capitalized banks, compared to the ones with high levels of capitalization, are less efficient. however, it shows that higher capitalized banks consistently keep more than 1% LLRs out of total loans.

## Introduction

Bank performance has been and will always be a pivotal issue as far as the government and banking regulatory authorities are concerned because of its significant contributions to the country’s economy through its intermediary role played between borrowers and lenders. China, as the largest developing country in the world, attaches even greater importance to the healthy development of its banking sector considering that the other three pillars in the financial system (the insurance industry, trust industry and stock market) are not well developed compared to those from the European countries. The Chinese government took efforts and engaged in a series of banking reforms to cope with the issues. The fundamental obstacle of Chinese banks at the beginning of the twenty-first century is the issue of non-performing loans. Therefore, the Chinese government injected capital to the Chinese banking sector to improve bank competitiveness. Over the period 2003–2008, a total of 57.5 billion USD and 162.5 billion RMB had been injected to several state-owned and joint-stock commercial banks including Bank of China, China Construction Bank, Industrial and Commercial Bank of China, Bank of Communication, China Everbright Bank and Agricultural Bank of China (Tan & Floros, 2018; Tan, 2016a). Another measurement taken by the Chinese government was the establishment of four asset management companies, the purpose of which was also to write-off the non-performing loans for the big state-owned banks. In addition, the Chinese government produced and implemented the policy of foreign strategic investment with the purpose of enhancing the practice of risk management for the joint-stock banks by allowing foreign banks to hold a number of shares from domestic banks. The operation of joint-stock banks became more stable thanks to the advanced risk management experience brought in by the foreign banks. Finally, the Chinese government established a banking regulatory authority in 2003, namely China Banking Regulatory Commission to enhance the prudential operation of Chinese commercial banks.^1^

The Chinese government introduced the loan classification system in 1998. This system classified the loans into 5 types according to their risk levels. Every loan type needs to set out a specific percentage of loan loss provisions (LLPs) to absorb the potential negative shock. The five types of loans are pass loans, special attention, substandard, doubtful and loss. The Chinese commercial banks do not set aside any LLPs for the pass loans, they will have 2% out of special attention loans as the LLPs, 25% out of substandard loans as LLPs, 50% out of doubtful loans as LLPs and 100% out of loss loans as LLPs. Besides the criteria illustrated above related to the special LLPs, Chinese commercial banks are required to hold certain amounts of LLRs by the end of the year, with the ratio of LLRs to total loans of no less than 1%.

The academic researchers have also consistently been taking efforts to investigate the issue of banking sector stability from the following perspectives: (1) the investigation of bank stability and bank performance (Bitar et al., 2016; Fang et al., 2019; Tan et al., 2017; Tan, 2016b; Zhang et al., 2013); (2) the evaluation of bank competition and bank risk (Albaity et al., 2019; Beck et al., 2013; Fu et al., 2014; Nguyen et al., 2012; Tan & Anchor, 2017); (3) the examination of bank stability and corporate governance (Bai & Elyasiani, 2013; Srivastav & Hagendorff, 2015; Laeven & Levine, 2009; Gaganis et al., 2020); and (4) the assessment of bank stability and bank ownership (Chalermchatvichien et al., 2014; Deng et al., 2013; Drakos et al., 2016; Lee & Hsieh, 2014; Pak, 2019).

One stream of studies, as mentioned above, focused its contributions on evaluating the relationship between bank stability and other variables concerned. In particular, the examination of the relationship between bank stability and bank performance concentrated on the issue of whether risk factor should be incorporated in the efficiency analysis (Chang & Chiu, 2006; Chiu et al., 2011; Delis et al., 2017) or whether risk should be separated from the efficiency analysis, and its relationship be tested in a second stage regression analysis (Fiordelisi, Marques-Ibanez, & Molynuex, 2011; Zhang et al., 2013; Konara et al., 2019). During the past decade, network Data Envelopment Analysis (NDEA) has been introduced and it has undergone significant development in the area of efficiency analysis applied in the banking industry (Fukuyama & Matousek, 2017; Wanke et al., 2019; among others). There is a common characteristic among all the studies; the intermediate outputs used in the network model mainly focus on bank-specific outputs, such as deposits, and loans are considered as the final output. Fukuyama and Tan (2020) proposed a three-stage NDEA to investigate three types of bank efficiencies, through which not only deposits were included as the intermediate products, but also they consider the deposit-based market power as another intermediate product, which affects the loan generation in the next stage, similarly, loan-based market power is considered to play a role in generating the final outputs including interest income and non-interest income.

Another interesting development in NDEA model applying to the banking sector is the use of non-performing loans in the modelling framework. Higher volumes of non-performing loans are not good for bank stability and banks make efforts to minimize the size and effect of this. In the banking sector, banks allocate credits to different sectors across the economy. Because of the unbalanced development among different industries, the risk level would be different among different sectors, which results in the generation and accumulation of non-performing loans. Therefore, the non-performing loans are to some extent non-avoidable, although scenarios exist for bank-year observations without non-performing loans. Banks have the mechanism to deal with the issue of non-performing loans by setting aside LLPs, through which to absorb potential negative shock and enhance bank stability. As discussed previously, the Chinese banking industry had a common LLRs to total loans ratio of 1% by the end of the year, according to the regulation. Therefore, due to the bank stability consideration, if the ratio is less than 1% which indicates that it is not in line with the regulation, we argue that the LLRs are not desirable. In other words, it is a bad output. On the other hand, if the ratio is more than 1% for some bank-year observations, we argue that it is a prudential operation and banks will be more stable, therefore, it can be regarded as a good output.

The current study makes significant contributions to the empirical literature incorporating risk factors in banking efficiency analysis from the following perspectives: (1) we are the first to consider using LLRs as a risk indicator with a dual role. To be more specific, we treat LLRs as a bad output if its ratio over total loans is less than 1%, whereas the LLRs will be treated as a desirable output if its ratio over total loans is over 1%. Therefore, we are the first to propose that “risk” in the banking production process is not necessarily bad but it can be good or bad; compared to the methods in the previous literature, our proposal and consideration benefits from the advantage that it is able to produce policy implications regarding the level of LLRs that should be held in order to achieve a higher level of efficiency (2) We implement the two features of the ratio in our DEA model using the costly disposability property presented by Murty et al. (2012) and Murty and Russell (forthcoming); compared to other efficiency studies that did not consider this property, our proposed method has the advantage of obtaining more accurate and robust results. (3) we compare the efficiency scores among three different scenarios: a. the efficiency scores with LLRs treated as good outputs; b. the efficiency scores with LLRs regarded as bad output; c. the efficiency scores without consideration of LLRs in the banking production process; the estimation of this would be helpful to generate the results showing the potential inflation or deflation of efficiency scores when risk factors are not considered in the production process, this has not been attempted by the previous studies;) we compare the level of efficiency under the above three scenarios according to the level of bank size, bank liquidity and equity capital. We generate the results regarding the impact of bank size, liquidity and capital on efficiency using only one-stage efficiency analysis and the results can be compared with other previous studies engaging in a second-stage regression analysis. In particular, we would be able to find out whether the impacts will be consistent across different scenarios with LLRs playing different roles in the production process.

The results show that the banks with the ratio between LLRs and total loans less than 1% have higher level of efficiency compared to the ones holding the ratio greater than 1%. The results show that when excluding LLRs in the production process, the efficiency scores are significantly inflated. We find that small and medium sized banks are more efficient than their big counterparts, however, the results show that big banks hold more than enough amounts of LLRs than the one required by the regulatory authority. When LLRs are excluded from the production process, it shows that big banks perform better than small and medium sized banks. Our findings show that less liquid banks perform better than the ones with higher levels of liquidity no matter in which way LLRs are treated. Finally, we find that lower capitalized banks, compared to the ones with high levels of capitalization, are less efficient. however, it shows that higher capitalized banks consistently keep more than 1% LLRs out of total loans. Our paper has the following structure: relevant bank efficiency studies incorporating risk factors in the models are reviewed in Sect. 2; Sect. 3 presents the model before the dataset is introduced in Sect. 4. Section 5 present the results. Finally, the discussions and conclusions are provided in Sect. 6.

## Literature review

In the previous section, we briefly talked about the four different types of research investigating banking sector stability, one of which was the examination of bank stability and bank performance. This topic can be further divided into two sub-evaluations according to the types of empirical analysis: one group of studies incorporated risk factors in the banking production process and relevant operational research methods have been applied to assess the level of bank efficiency, whereas another group of studies did not incorporate the risk factors in the production process but examined the impact or inter-relationships between bank stability and bank performance in a second stage regression analysis. Because the current paper is a piece of operational research in bank efficiency analysis, we are going to review the empirical literature focusing on the operational research in bank efficiency that incorporated risk factors in the analysis.

Looking at the most recent 10 years empirical literature, it is not difficult to observe that the volumes of empirical operational research in banking efficiency incorporating risk factors in the production process have grown tremendously. For the Japanese banking industry between 2000 and 2007, the technical efficiency is evaluated by Barros et al. (2012) with consideration of non-performing loans as bad outputs under a Russell Directional Distance Function.^2^ The results show that non-performing loans significantly and negatively influence bank performance. They further argue that Japanese banks should utilise the inputs such as labour and premises in a more efficient way. Similar research was also conducted by Fujii et al. (2014). They further apply the model to measure total factor productivity change with the incorporation of non-performing loans in the production process. The findings exhibit that the performance of different ownership types various and foreign ownership would facilitate the efficiency improvement. Rather than using the method proposed by Fujii et al. (2014), Puri and Yadav (2014) argue that, because the input and output data are not always available in the desirable form, the traditional DEA methods would be unable to cope with this issue. Therefore, a DEA model incorporating undesirable outputs (non-performing assets) has been analysed in a fuzzy environment. The findings suggest that the increase/decrease in the efficiency level comes with the same pattern as the increase/decrease in the non-performing assets ratio.

Instead of using DEA models to incorporate risk factors in the efficiency analysis, the non-performing loans is incorporated in an input distance function and the efficiency, productivity growth and efficiency growth are estimated in the Turkish banking industry during 2002–2010 under a Bayesian stochastic frontier analysis by Assaf et al. (2013). The findings indicate that productivity growth is positive, which is mainly attributed to technological improvement, while the efficiency growth is noticed to be negative and the results suggest that incorporating non-performing loans in the banking production process will distort the results of bank efficiency and bank productivity. Using Matthew’s (2013) data, Lozano (2016) distinguishes from Fukuyama and Weber (2010) by estimating bank efficiency under a general network model with incorporation of bad outputs. The target values of inputs, outputs and intermediate products have also been provided. The findings suggest that not only the source of inefficiency could be identified by the linear programming general network model, but also the overall network slacks-based inefficiency scores as well as the scores of different processes and target values of each decision-making unit can be estimated. A revenue function incorporating non-performing loans is developed by Fukuyama and Matousek (2017) to estimate the revenue efficiency in the Japanese banking industry between 2000 and 2013. In addition, the authors applied Nerlove’s revenue indicator, from which both the output and allocative inefficiencies can be disaggregated. The findings suggest that the Japanese banking industry did not achieve the optimal level of production. The results further recommend that the Japanese banks should expand the output production, including securities and other earning assets as well as loans. For the Bangladesh banking industry, Akther et al. (2013) estimate the slacks-based inefficiency under a two-stage network model. The method is based on the one used by Fukuyama and Weber (2010), while it is further extended by considering the way that the first period bad loans constrain the production possibilities in the next period. The findings suggest that efficiency can be improved in the first stage by expanding the deposits production and inefficient banks would be able to expand the production of desirable outputs, including loans and securities while reducing the production of bad loans.

There are few studies investigating the Chinese commercial banks that incorporate risk factors in the efficiency analysis. An et al. (2015) evaluate the slacks-based efficiency over the period 2008–2012 by dividing the production process into two-stages: the deposit generation stage and the deposit utilization stage. The analysis incorporated bad loans as the undesirable outputs. The results from the two-stage network analysis show that the improvement in the deposit-utilization stage contributes more to the overall improvement in the efficiency level compared to the deposit generation stage. A similar type of research has also been conducted by Wang et al. (2014). Another interesting piece of research from Matthews (2013) analyses Chinese bank efficiency under a three-stage DEA with the consideration of bad output (non-performing loans). This piece of research differs from other empirical literature by using qualitative data in estimating efficiency. Two metrics have been constructed from questionnaires related to risk management practice and risk management organization, both of which were used as intermediate inputs to produce the desirable output (interest earnings) and undesirable outputs (non-performing loans). The results suggest that there is no direct relationship between this constructed performance measurement and the direct performance indicator, such as Return on Assets, while the efficiency scores generated when incorporating these two intermediate inputs explain the return on assets in a better way compared to the ones that exclude them.

Zha et al. (2016) evaluate Chinese bank efficiency level between 2008 and 2012 under a network two-stage DEA with consideration of the carry-over character of non-performing loans. The production process has been divided into productivity stage, where operating costs and interest costs are used to produce deposit, while deposit as an intermediate product is used as the input in the profitability stage to generate interest and operation income. For a given period of time, the non-performing loans are the undesirable outputs and undesirable inputs in the profitability stage and the productivity stage, respectively. The results show that technical and scale inefficiencies are derived from both the productivity stage and the profitability stage.

The LLPs are firstly considered in the efficiency analysis by Tsolas and Charles (2015). The LLPs and haircut losses on Greek bonds are regarded as inputs, with the former carrying a stochastic controllable characteristic and the latter uncontrollable, A satisficing DEA combined with a Monte Carlo simulation is used to estimate the efficiency score. Both strong and weak disposability assumptions are made in terms of the undesirable output. The findings suggest that the non-core banks perform better than the core banks. In particular, the study argues that the method proposed in the study is able to provide the possibility of a decision-making unit to be efficient for predefined aspiration levels, while the deterministic DEA model is unable to achieve this. The results derived from the study are able to provide more accurate policy implications.

Most recently, a general multi-stage structure- data envelopment analysis model is proposed by Tan et al. (2021), in which LLPs are treated as inputs to generate the intermediate product, through which the final outputs are produced. The proposed method benefits from the advantages of being able to relax the assumption that exogenous inputs are not consumed and exogenous outputs are not produced during the internal processes. The findings suggest that the Chinese banking industry are more efficient in generating income related final outputs and less efficiency in producing the intermediate products.

LLPs are considered as the outputs in estimating stability efficiency in the Chinese banking industry by Tan and Tsionas (2020). Under the proposal of using total deposits, fixed assets, number of employees as well as the fixed input equity capital, they also estimate another three types of efficiencies including social efficiency, environmental efficiency and economic efficiency. The integration of these four sub-efficiencies make up the overall sustainability efficiency. The findings from the output distance function and the panel vector autoregression model show that Chinese commercial banks have a big different in the level of stability efficiency and the scores range from 0.575 to 0.855, while the overall sustainability efficiency ranges between 0.45 and 0.75.

Not only LLPs, but the empirical studies have also tried to incorporate LLRs^3^ in bank efficiency estimation, one of which is conducted by Liu and Tone (2008) who propose a multistage method for efficiency analysis in the Japanese banking industry. The first stage uses weighted slacks-based model focusing on slacks, the second stage uses stochastic frontier analysis focusing on decomposition and final stage uses weighted slacks-based model again focusing on efficiency score and ranks. Three inputs are considered including interest expenses, credit cost and general and administrative expenses, while two outputs are considered including interest-accruing loans and lending revenue. LLRs are included in the credit cost.

Another piece of research attempts to use LLRs^4^ as an undesirable carry-over input in a dynamic slacks-based model. This is conducted by Wanke et al. (2015) in the Brazilian banking context. The results show that small national and public banks are more inefficient. The study further shows that size, mergers and acquisitions are important for bank efficiency and foreign banks do not contribute to the efficiency improvement. Finally, an attempt has been made to incorporate both non-performing loans and LLRs in the banking production process. Chao et al. (2015) propose a three-stage NDEA to evaluate the efficiency level using a sample of Taiwanese banks. On top of its innovation to define intellectual capital creation capacity as the first stage of the production process, followed by efficiency stage (generate loans and investments) and profitability stage (generate interest income, fee income and other investment-related income), they define both non-performing loans and LLRs as the carryover items.

As we can see from the review of the empirical literature above, different advanced operational methods have been applied to the banking industry to estimate the efficiency level with consideration of non-performing loans in the production process. Few attempts have been made to incorporate LLPs and LLRs in the banking production process to estimate bank efficiency. However, these few studies are lack of careful consideration. More specifically, the main purpose for banks to set aside LLPs every year is to absorb the potential losses derived from the non-performing loans. The government and banking regulatory authority paid special attention to this due to the fact that bank instability derived from lack of LLPs will have a devastating effect on the whole financial system and further on the whole economy. Therefore, for the Chinese banking industry specifically, in order to minimize the negative effect, the banking regulatory authority set up a threshold for all the banks to keep as the minimum requirement. In the Chinese case, it is required that all the banks should keep at least 1% out of total loans as the LLRs. However, as the data shows that some banks at some years kept less than 1% LLRs, while this was related to 1) banks’ own evaluations on the risk levels of the loan portfolio, i.e. if banks think that the quality of the loan portfolio is very good, they will set aside less than 1% LLPs, this will lead to a possibility that the level of LLRs accumulated from LLPs will be less than 1% of the total loans 2) LLRs will obviously reduce the level of bank profitability. The banks should abide by this recommendation for stability purpose. Therefore, for those banks at specific years with less than 1% LLRs, the LLRs would be regarded as undesirable outputs, otherwise, they would be treated as desirable outputs. The empirical studies failed to consider this, and the current study is the first one to treat the LLRs with a dual role.

## Model

In this study we utilize a three-stage NDEA. In Stage 1 a bank uses various inputs ${\mathbf{x}} \in \Re_{ + }^{N}$ to produce the intermediate products ${\mathbf{z}}_{{}}^{(1,2)} \in \Re_{ + }^{{Q_{{}}^{(1,2)} }}$. This intermediate products are used as inputs of Stage 2 to produce another set of intermediate products ${\mathbf{z}}_{{}}^{(2,3)} \in \Re_{ + }^{{Q_{{}}^{(2,3)} }}$, which is consumed to generate the intended final outputs ${\mathbf{y}} \in \Re_{ + }^{M}$ in Stage 3. In this case the standard three-stage production possibility set can be stated as follows:

1 $$ \left\{ {\left( {{\mathbf{x}},{\mathbf{z}}_{{}}^{(1,2)} ,{\mathbf{z}}_{{}}^{(2,3)} ,{\mathbf{y}}} \right)\left| \begin{gathered} \;{\mathbf{z}}_{{}}^{(1,2)} {\text{ is producible from }}{\mathbf{x}}\quad ({\text{Stage }}1) \hfill \\ \;{\mathbf{z}}_{{}}^{(2,3)} {\text{ is producible from }}{\mathbf{z}}_{{}}^{(1,2)} \quad ({\text{Stage }}2) \hfill \\ \;{\mathbf{y}}{\text{ is producible from }}{\mathbf{z}}_{{}}^{(2,3)} \quad ({\text{Stage }}3) \hfill \\ \end{gathered} \right.} \right\} $$

**Fig. 1:**
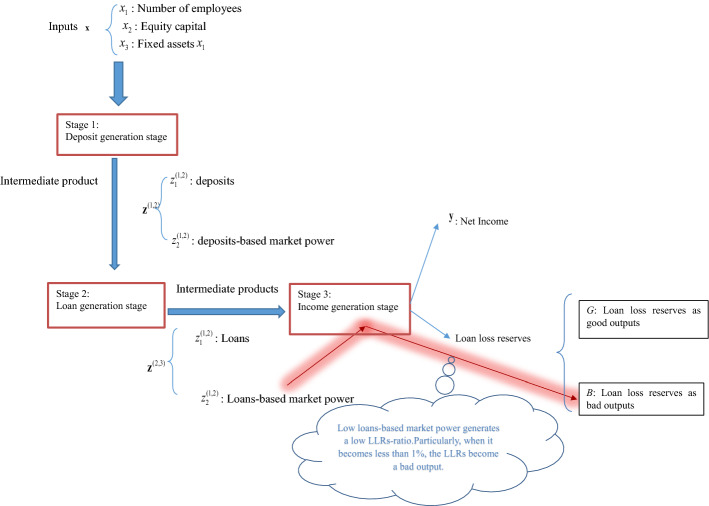
The dual role of loan loss reserves in banking production process

In this paper we extend the standard three-stage technology (1) to the one which reflects Chinese bank behaviour. In Fig. 1, production of a Chinese bank is assumed to have three-stage production processes, where.

$$ \begin{gathered}   {\mathbf{x}} = (x_{1} ,x_{2} ,x_{3} ) = ({\text{labor,}}\;{\text{equity capital,fixed assets}}), \hfill \\   {\mathbf{z}}^{{(1,2)}}  = (z_{1}^{{(1,2)}} ,z_{2}^{{(1,2)}} ) = ({\text{deposits, deposits-based market power}}), \hfill \\   {\mathbf{z}}^{{(2,3)}}  = (z_{1}^{{(2,3)}} ,z_{2}^{{(2,3)}} ) = ({\text{loans, loans-based market power}}) \hfill \\  \end{gathered}  $$

y = (Net income), with an additional good output $G \in \Re_{ + }^{{}}$ and a bad (undesirable) output $B \in \Re_{ + }^{{}}$.

We use personnel expenses, equity capital and fixed assets as the inputs in the first stage following Sturn and Williams (2004) and Antunes et al., (2021). The proposal of using deposits, deposit-based market power, loans as well as loan-based market power is in line with Fukuyama and Tan (2020). Due to the consideration that the ultimate goal for the banking operation is to generate income, therefore, we propose to use net income as the final output following Fukuyama and Tan (2021a).

In this study the ratio between LLRs and total loans (LLRs-ratio) is used as an output. The bigger its value, the more stable the bank will be, but if it is too low then it will face risk of bankruptcy. As a consequence, the ratio has two characteristics: desirable and undesirable features, i.e., we label it as either a good output or a bad output, depending upon whether it is greater than or less than 1%. We use 1% as the value which distinguishes from good output to bad output. That is, we differentiate the LLRs-ratio values as follows:

2 $$ \begin{gathered} {\text{LLRs-ratio }} \ge 1{\text{\% }}\quad \Rightarrow \quad G\;\left( {\text{LLRs related good output}} \right) \hfill \\ {\text{ LLRs-ratio }} < 1{\text{\% }}\quad \Rightarrow \quad B\;\left( {\text{LLRs related bad output}} \right) \hfill \\ \end{gathered} $$

The empirical banking literature has documented the relationship between competition and risk through two different hypotheses, namely, competition-stability hypothesis and competition-instability hypothesis. Boyd and De Nicole (2005) argue that higher interest rate will be charged by banks under a lower competitive environment, and this will result in an increase in the loan repayment default. On the other hand, the competition-instability theory suggests that the risk level will be lower because a lower level of competition will increase bank profitability and banks are more capable of absorbing losses.

The total amount of loans ($z_{1}^{(2,3)}$), as it is, does not make the LLRs-ratio a bad output, but the loans-based market power directly does. The LRRs-ratio, as an important indicator of bank risk, is affected by banking sector competition measured by the loan-based market power. This relationship is well documented in the competition stability and competition fragility hypotheses (Tan, 2014; Tan & Floros, 2019). Therefore, the LLRs-ratio is directly affected by loans-based market power $z_{2}^{(2,3)}$. We model this characteristic according to Murty et al.’s (2012) by-production approach. The resulting overall technology is expressed as follows:

3 $$ \left\{ {\left( {{\mathbf{x}},{\mathbf{z}}_{{}}^{(1,2)} ,{\mathbf{z}}_{{}}^{(2,3)} ,{\mathbf{y}},G,B} \right)\left| \begin{gathered} \;{\mathbf{z}}_{{}}^{(1,2)} {\text{ is producible from }}{\mathbf{x}}\quad ({\text{Stage }}1) \hfill \\ \;{\mathbf{z}}_{{}}^{(2,3)} {\text{ is producible from }}{\mathbf{z}}_{{}}^{(1,2)} \quad ({\text{Stage 2}}) \hfill \\ \;({\mathbf{y}},G,B){\text{ is producible from }}{\mathbf{z}}_{{}}^{(2,3)} \quad ({\text{Stage 3 - 1}}) \hfill \\ \;(G,B){\text{ is producible from }}{\mathbf{z}}_{{}}^{(2,3)} \quad ({\text{Stage 3 - 2)}} \hfill \\ \end{gathered} \right.} \right\} $$

with costly disposability with respect to $z_{2}^{(2,3)}$ and B:

4 $$ \begin{gathered} \left( {{\mathbf{x}},z_{1}^{(1,2)} ,z_{2}^{(1,2)} ,z_{1}^{(2,3)} ,z_{2}^{(2,3)} ,{\mathbf{y}},G,B} \right) \in T\quad \wedge \quad \hat{z}_{2}^{(2,3)} \le z_{2}^{(2,3)} \quad \wedge \quad \hat{B} \ge B \hfill \\ \quad \quad \quad \Rightarrow \quad \left( {{\mathbf{x}},z_{1}^{(1,2)} ,z_{2}^{(1,2)} ,z_{1}^{(2,3)} ,\hat{z}_{2}^{(2,3)} ,{\mathbf{y}},G,\hat{B}} \right) \in T \hfill \\ \end{gathered} $$

Murty et al. (2012) developed the costly disposability property as a joint property between emission-causing input and emissions. The term costly disposability was first used by Murty and Russell (forthcoming). Related to the costly disposability property, Ray and Mukherjee (2007) considered a weakly disposable technology (Ray et al. (2018)). See also Färe and Grosskopf (2009) and Dakpo et al. (2016) for some discussions on various disposability properties and unintended outputs.

For a bank $j = 1,...,J$, let $\left( {{\mathbf{x}}_{j}^{{}} ,z_{1j}^{(1,2)} ,z_{2j}^{(1,2)} ,z_{1j}^{(2,3)} ,z_{2j}^{(2,3)} ,{\mathbf{y}}_{j}^{{}} ,G_{j}^{{}} ,B_{j}^{{}} } \right)$ represent the observed production vector. Let $\lambda_{j}^{k} \;\left( {j = 1,...,J} \right)$ be the intensity variables of stage $k = 1,2,3$. Using the observations, we construct various internal technologies as follows:

Stage 1 technology:

5 $$ T_{{}}^{1} = \left\{ {\left( {{\mathbf{x}},{\mathbf{z}}_{{}}^{(1,2)} } \right)\left| {\;{\mathbf{x}} \ge \sum\nolimits_{j = 1}^{J} {{\mathbf{x}}_{j}^{{}} \lambda_{j}^{1} } ,\;\;{\mathbf{z}}_{{}}^{(1,2)} \le \sum\nolimits_{j = 1}^{J} {{\mathbf{z}}_{j}^{(1,2)} \lambda_{j}^{1} } ,\;\;\sum\nolimits_{j = 1}^{J} {\lambda_{j}^{1} } = 1,\;\;\lambda_{j}^{1} \ge 0\;\;\forall j.} \right.} \right\} $$

Stage 2 technology:

6 $$ T^{2} = \left\{ {\left( {{\mathbf{z}}^{(1,2)} ,{\mathbf{z}}^{(2,3)} } \right)\left| {\;{\mathbf{z}}^{(1,2)} \ge \sum\nolimits_{j = 1}^{J} {{\mathbf{z}}_{j}^{(1,2)} \lambda_{j}^{2} } ,\;\;{\mathbf{z}}^{(2,3)} \le \sum\nolimits_{j = 1}^{J} {{\mathbf{z}}_{j}^{(2,3)} \lambda_{j}^{2} } ,\;\;\sum\nolimits_{j = 1}^{J} {\lambda_{j}^{2} } = 1,\;\;\lambda_{j}^{2} \ge 0\;\;\forall j.} \right.} \right\} $$

If the LLP-ratio is treated as a good output, then Stage 3 technology is shown as follows.

7 $$ G - T^{3} = \left\{ {\left( {{\mathbf{z}}^{(2,3)} ,\;{\mathbf{y}},G,B} \right)\left| {\begin{array}{*{20}l} {{\mathbf{z}}^{(2,3)} \ge \sum\limits_{j = 1}^{J} {{\mathbf{z}}_{j}^{(2,3)} }_{j} \lambda_{j}^{3} ,\;{\mathbf{y}} \le \sum\limits_{j = 1}^{J} {{\mathbf{y}}_{j} \lambda_{j}^{3 - 1} } ,\;\;G \le \sum\limits_{j = 1}^{J} {G_{j} \lambda_{j}^{3} } ,\;} \hfill \\ {\;\sum\limits_{j = 1}^{J} {\lambda_{j}^{3} } = 1,\;\lambda_{j}^{3} \ge 0\;\;\forall j.} \hfill \\ \end{array} } \right.} \right\} $$

If the LLRs-ratio is considered as a bad output, then Stage 3 technology consists of intended sub-technology and residual generation technology (Murty et al., 2012).

Stage 3 sub-technology 1 (intended good production technology);

8 $$ B - T^{3 - 1} = \left\{ {\left( {z^{(2,3)} ,y,G,B} \right)\left| \begin{gathered} \;{\mathbf{z}}^{(2,3)} \ge \sum\nolimits_{j = 1}^{J} {{\mathbf{z}}_{j}^{(2,3)} }_{j} \lambda_{j}^{3 - 1} ,\;\;y \le \sum\nolimits_{j = 1}^{J} {y_{j} \lambda_{j}^{3 - 1} } ,\; \hfill \\ \;\sum\nolimits_{j = 1}^{J} {\lambda_{j}^{3 - 1} } = 1,\;\;\lambda_{j}^{3 - 1} \ge 0\;\;\forall j. \hfill \\ \end{gathered} \right.} \right\} $$

Stage 3 sub-technology 2 (residual bad output generation technology);

9 $$ B{ - }T^{3 - 2} = \left\{ {\left( {{\mathbf{z}}_{2}^{(2,3)} ,{\mathbf{y}},G,B} \right)\left| \begin{gathered} \;{\mathbf{z}}_{2}^{(2,3)} \le \sum\nolimits_{j = 1}^{J} {{\mathbf{z}}_{2j}^{(2,3)} }_{j} \lambda_{j}^{3 - 2} ,\;\;B \ge \sum\nolimits_{j = 1}^{J} {B_{j} \lambda_{j}^{3 - 2} } ,\; \hfill \\ \;\sum\nolimits_{j = 1}^{J} {\lambda_{j}^{3 - 2} } = 1,\;\;\lambda_{j}^{3 - 2} \ge 0\;\;\forall j. \hfill \\ \end{gathered} \right.} \right\} $$

Stage 3 technology consists of (8) and (9), i.e., it is the intersection of $$ B{-}T^{3-1}$$ and $$B{-}T^{3-2}$$. The overall by-production production possibility set consisting of (5), (6), (7), (8) and (9) is constructed by

10 $$T=\left\{\left(\begin{array}{c}x\\ {{\varvec{z}}}^{\left(\mathrm{1,2}\right)}\\ {{\varvec{z}}}^{\left(\mathrm{2,3}\right)}\\ y\\ G\\ B\end{array}\right)\left|\begin{array}{c}\hspace{0.33em}x\ge {\sum }_{j=1}^{J}{{\varvec{x}}}_{j}{\lambda }_{j}^{1},\hspace{0.33em}\hspace{0.33em}{{\varvec{z}}}^{\left(\mathrm{1,2}\right)}\le {\sum }_{j=1}^{J}{{\varvec{z}}}_{j}^{\left(\mathrm{1,2}\right)}{\lambda }_{j}^{1},\hspace{0.33em}\hspace{0.33em}{\sum }_{j=1}^{J}{\lambda }_{j}^{1}=1,\hspace{0.33em}\hspace{0.33em}{\lambda }_{j}^{1}\ge 0\hspace{0.33em}\hspace{0.33em}\forall j,\hspace{0.33em}\\ \hspace{0.33em}{{\varvec{z}}}^{\left(\mathrm{1,2}\right)}\ge {\sum }_{j=1}^{J}{{{\varvec{z}}}^{\left(\mathrm{1,2}\right)}}_{j}{\lambda }_{j}^{2},\hspace{0.33em}\hspace{0.33em}{{\varvec{z}}}^{\left(\mathrm{2,3}\right)}\le {\sum }_{j=1}^{J}{{\varvec{z}}}_{j}^{\left(\mathrm{2,3}\right)}{\lambda }_{j}^{2},\hspace{0.33em}\hspace{0.33em}{\sum }_{j=1}^{J}{\lambda }_{j}^{2}=1,\hspace{0.33em}\hspace{0.33em}{\lambda }_{j}^{2}\ge 0\hspace{0.33em}\hspace{0.33em}\forall j,\\ \hspace{0.33em}{{\varvec{z}}}^{\left(\mathrm{2,3}\right)}\ge {\sum }_{j=1}^{J}{{{\varvec{z}}}_{j}^{\left(\mathrm{2,3}\right)}}_{j}{\lambda }_{j}^{3-1},\hspace{0.33em}\hspace{0.33em}y\le {\sum }_{j=1}^{J}{{\varvec{y}}}_{j}{\lambda }_{j}^{3-1},\hspace{0.33em}\hspace{0.33em}{\sum }_{j=1}^{J}{\lambda }_{j}^{3-1}=1,\hspace{0.33em}\hspace{0.33em}{\lambda }_{j}^{3-1}\ge 0\hspace{0.33em}\hspace{0.33em}\forall j,\hspace{0.33em}\\ \hspace{0.33em}{z}_{2}^{\left(\mathrm{2,3}\right)}\le {\sum }_{j=1}^{J}{{z}_{2j}^{\left(\mathrm{2,3}\right)}}_{j}{\lambda }_{j}^{3-2},\hspace{0.33em}\hspace{0.33em}{\sum }_{j=1}^{J}{\lambda }_{j}^{3-2}=1,\hspace{0.33em}\hspace{0.33em}{\lambda }_{j}^{3-2}\ge 0\hspace{0.33em}\hspace{0.33em}\forall j,\\ \hspace{0.33em}G\le {\sum }_{j=1}^{J}{G}_{j}{\lambda }_{j}^{3-1},\hspace{0.33em}\hspace{1em}\hspace{1em}\hspace{1em}{\text{i}}{\text{f}} \, \, LLRs-{\text{r}}{\text{a}}{\text{t}}{\text{i}}{\text{o}} \, {\text{i}}{\text{s}} \, {\text{a}} \, {\text{good}} \, {\text{output}}\\ \hspace{0.33em}B\ge {\sum }_{j=1}^{J}{B}_{j}{\lambda }_{j}^{3-2},\hspace{0.33em}\hspace{1em}\hspace{1em}\hspace{1em}{\text{i}}{\text{f}} \, \, LLRs-{\text{r}}{\text{a}}{\text{t}}{\text{i}}{\text{o}} \, {\text{i}}{\text{s}} \, {\text{a}} \, {\text{bad}} \, {\text{output}}\end{array}\right.\right\}$$

The intended and unintended outputs can be jointly produced with an overall technology by means of two sub-technologies: intended technology and residual-generation technology (Førsund, 2009, 2018).

We formulate two linear programming problems. If the LLRs-ratio is a good output according to (2), a slack-based inefficiency G measure is defined as:

11 $$ \begin{gathered} G{ - }NSBI\left( {{\mathbf{x}},\;{\mathbf{y}},G;\;{\mathbf{g}}} \right) \hfill \\ = \max \left\{ {\frac{1}{3}\left( \begin{gathered} \frac{1}{N}\sum\limits_{n = 1}^{N} {\frac{{s_{n}^{x} }}{{g_{n}^{x} }}} \hfill \\ + \frac{1}{M}\sum\limits_{m = 1}^{M} {\frac{{s_{m}^{y} }}{{g_{m}^{y} }}} \hfill \\ + \frac{{s^{G} }}{{g^{G} }} \hfill \\ \end{gathered} \right)\left| \begin{gathered} \;{\mathbf{x}} - {\mathbf{s}}^{x} \ge \sum\nolimits_{j = 1}^{J} {{\mathbf{x}}_{j} \lambda_{j}^{1} } ,\;\;{\mathbf{z}}^{(1,2)} \le \sum\nolimits_{j = 1}^{J} {{\mathbf{z}}_{j}^{(1,2)} \lambda_{j}^{1} } ,\;\;\sum\nolimits_{j = 1}^{J} {\lambda_{j}^{1} } = 1,\;\;\lambda_{j}^{1} \ge 0\;\;\forall j,\; \hfill \\ \;{\mathbf{z}}^{(1,2)} \ge \sum\nolimits_{j = 1}^{J} {{\mathbf{z}}^{(1,2)} }_{j} \lambda_{j}^{2} ,\;\;{\mathbf{z}}^{(2,3)} \le \sum\nolimits_{j = 1}^{J} {{\mathbf{z}}_{j}^{(2,3)} \lambda_{j}^{2} } ,\;\;\sum\nolimits_{j = 1}^{J} {\lambda_{j}^{2} } = 1,\;\;\lambda_{j}^{2} \ge 0\;\;\forall j, \hfill \\ \;{\mathbf{z}}^{(2,3)} \ge \sum\nolimits_{j = 1}^{J} {{\mathbf{z}}_{j}^{(2,3)} }_{j} \lambda_{j}^{3 - 1} ,\;\;{\mathbf{y}} + {\mathbf{s}}^{y} \le \sum\nolimits_{j = 1}^{J} {{\mathbf{y}}_{j} \lambda_{j}^{3 - 1} } ,\;\;G + s^{G} \le \sum\nolimits_{j = 1}^{J} {G_{j} \lambda_{j}^{3 - 1} } ,\; \hfill \\ \;\sum\nolimits_{j = 1}^{J} {\lambda_{j}^{3 - 1} } = 1,\;\;\lambda_{j}^{3 - 1} \ge 0\;\;\forall j,\;\;{\mathbf{z}}_{2}^{(2,3)} \le \sum\nolimits_{j = 1}^{J} {{\mathbf{z}}_{2j}^{(2,3)} }_{j} \lambda_{j}^{3 - 2} ,\; \hfill \\ \;\sum\nolimits_{j = 1}^{J} {\lambda_{j}^{3 - 2} } = 1,\;\;\lambda_{j}^{3 - 2} \ge 0\;\;\forall j,\;\;{\mathbf{z}}^{(1,2)} \ge {\mathbf{0}},\;\;{\mathbf{z}}^{(2,3)} \ge {\mathbf{0}} \hfill \\ \end{gathered} \right.} \right\} \hfill \\ \end{gathered} $$

If the LLRs-ratio is treated as a bad output, then a slack-based inefficiency B-measure is defined as:

12 $$ \begin{gathered} B{ - }NSBI\left( {{\mathbf{x}},\;{\mathbf{y}},B;\;{\mathbf{g}}} \right) \hfill \\ = \max \left\{ {\frac{1}{3}\left( \begin{gathered} \frac{1}{N}\sum\limits_{n = 1}^{N} {\frac{{s_{n}^{x} }}{{g_{n}^{x} }}} \hfill \\ + \frac{1}{M}\sum\limits_{m = 1}^{M} {\frac{{s_{m}^{y} }}{{g_{m}^{y} }}} \hfill \\ + \frac{{s^{B} }}{{g^{B} }} \hfill \\ \end{gathered} \right)\left| \begin{gathered} \;{\mathbf{x}} - {\mathbf{s}}^{x} \ge \sum\nolimits_{j = 1}^{J} {{\mathbf{x}}_{j} \lambda_{j}^{1} } ,\;\;{\mathbf{z}}^{(1,2)} \le \sum\nolimits_{j = 1}^{J} {{\mathbf{z}}_{j}^{(1,2)} \lambda_{j}^{1} } ,\;\;\sum\nolimits_{j = 1}^{J} {\lambda_{j}^{1} } = 1,\;\;\lambda_{j}^{1} \ge 0\;\;\forall j,\; \hfill \\ \;{\mathbf{z}}^{(1,2)} \ge \sum\nolimits_{j = 1}^{J} {{\mathbf{z}}^{(1,2)} }_{j} \lambda_{j}^{2} ,\;\;{\mathbf{z}}^{(2,3)} \le \sum\nolimits_{j = 1}^{J} {{\mathbf{z}}_{j}^{(2,3)} \lambda_{j}^{2} } ,\;\;\sum\nolimits_{j = 1}^{J} {\lambda_{j}^{2} } = 1,\;\;\lambda_{j}^{2} \ge 0\;\;\forall j, \hfill \\ \;{\mathbf{z}}^{(2,3)} \ge \sum\nolimits_{j = 1}^{J} {{\mathbf{z}}_{j}^{(2,3)} }_{j} \lambda_{j}^{3 - 1} ,\;\;{\mathbf{y}} + {\mathbf{s}}^{y} \le \sum\nolimits_{j = 1}^{J} {{\mathbf{y}}_{j} \lambda_{j}^{3 - 1} } ,\;\;\sum\nolimits_{j = 1}^{J} {\lambda_{j}^{3 - 1} } = 1,\; \hfill \\ \;\lambda_{j}^{3 - 1} \ge 0\;\;\forall j,\;\;z_{2}^{(2,3)} \le \sum\nolimits_{j = 1}^{J} {z_{2j}^{(2,3)} }_{j} \lambda_{j}^{3 - 2} ,\;\;B - s^{B} \ge \sum\nolimits_{j = 1}^{J} {B_{j} \lambda_{j}^{3 - 2} } ,\; \hfill \\ \;\sum\nolimits_{j = 1}^{J} {\lambda_{j}^{3 - 2} } = 1,\;\;\lambda_{j}^{3 - 2} \ge 0\;\;\forall j,\;\;{\mathbf{z}}_{{}}^{(1,2)} \ge {\mathbf{0}},\;\;{\mathbf{z}}^{(2,3)} \ge {\mathbf{0}} \hfill \\ \end{gathered} \right.} \right\} \hfill \\ \end{gathered} $$

The details of black box and network SBI models can be found in Fukuyama and Weber (2009) and Fukuyama and Weber (2010). For a comparison purpose, Appendix 1 presents ${\text{std - }}NSBI\left( {{\mathbf{x}},\;{\mathbf{y}};\;{\mathbf{g}}} \right)$, which does not include the LLRs index. I.e., ${\text{std - }}NSBI\left( {{\mathbf{x}},\;{\mathbf{y}};\;{\mathbf{g}}} \right)$ is constructed without consideration of LLRs.

## Dataset

The current paper collects a dataset with 43 banks in the Chinese banking sector between 2010–2019. We choose this time period for the estimation is mainly attributed to the consideration that the shock derived from external environment would distort the banking statistics and further influence the results, therefore we exclude the period of global financial crisis between 2007 and 2009 as well as the Covid-19 that was outbroken in 2020, however, we cover the whole period of 10 years across 2010 to 2019. The data is collected from the Fitchconnect database, which provides annual financial statements of more than 30,000 public and private banks across the globe.

We use a three-stage network DEA model incorporating and considering the dual role of LLRs in the banking production process. In terms of the inputs and outputs selection in production process, there are mainly two approaches widely used, one is the intermediation approach, which posits that banks use deposits and other purchased funds to generate loans and other assets, whereas the production approach assumes that banks are service providers for account holder and borrowers. In the current study, we follow and build on the intermediation approach adopted by (Fukuyama & Matousek, 2011, 2017). In summary, our production process is as below: banks use three inputs in the first stage including (1) personnel expenses; (2) equity capital; and (3) fixed assets. The banks use these three original inputs to produce two intermediate outputs which are: (1) deposits; (2) deposits-based market power. Following Fukuyama and Tan (2020), we measure the level of market power or market share occupied by dividing the amount of deposits by the total amount of deposits in the sample for that year. Higher values indicate higher market shares and higher market power. This stage (the first stage) is called deposit generation stage.

The two “intermediate” outputs, deposits and deposits-based market power will be used as the intermediate inputs in the second stage production. The banks will use these two “intermediate inputs” to generate another two “intermediate outputs”. They are: (1) loans; (2) loan-based market power. We measure the level of market power or market share occupied by a specific bank at a specific year by dividing the amount of loans by the total amount of loans in the sample for that year. Higher values indicate higher levels of market power, this is in line with Fukuyama and Tan (2020). This stage (the second stage) is called loan generation stage.

In the final income generation stage, the two intermediate outputs loans and loan-based market power will be used as the intermediate inputs to produce two kinds of final outputs: good/desirable outputs and bad/undesirable outputs. Net income is considered as the good/desirable outputs. Another output produced in the final stage is LLRs, which we argue may have a dual role as either a good/desirable output or a bad/undesirable output. The Chinese commercial banks were required to hold 1% loans by the end of the year as LLRs for the purpose of prudential operation. However, this was not the case when we analysed the data in the sample. More specifically, we find that some banks have an LLRs over total loan ratio of over 1% in some years, while in other years, the ratio was less than 1%. Thus, we consider the LLRs as good/desirable output for the bank-year observations with LLRs over total loan ratio of over 1%. In comparison, if a specific bank at a specific year holds an LLRs over total loans ratio of less than 1%, that suggests the bank would be in a risky position, therefore, we regard the LLRs as an undesirable/bad output. Finally, in our network three-stage DEA model, we assume the costly disposability is between loan-based market power and undesirable LLRs. We argue that lower market power in the loan market (higher level of competition) will make banks less risky. This accords with the competition-stability hypothesis and this is directly related to the undesirable LLRs. We implement this using the costly disposability property presented by Murty et al. (2012).

The descriptive statistics of inputs and outputs are provided in Appendix 1. Regarding the initial inputs, the statistics report there is a largest difference in the level of equity capital among the banks in the sample; although fixed assets are various in size, it is reported that there is a smaller difference in terms of personnel expenses compared to the other two inputs. The intermediate outputs in the deposit generation stage report that there is a huge difference in the level of deposits held; however, it is interesting to notice that the difference in market power among Chinese commercial banks is not big. This is also the case for the loan-based market power in the loan generation stage. It is observed that there is a big difference in the loan businesses engaged in, but the difference is smaller than the one of deposits. Finally, in the income generation stage, the ability of the Chinese banks to earn net income still varies to a large extent and the difference regarding the level of LLRs held is bigger compared to the one of net income.

## Empirical results

Recalling the model proposed in this paper, we implement the two features (desirable output and undesirable output) of the LLRs over total loans ratio in our DEA model using the costly disposability property presented by Murty et al. (2012). To be more specific, we assume that the cost disposability is between market power in loans and the undesirable LLRs over total loans ratio. Although there are two different theories regarding the effect of competition on stability in the banking sector, namely competition-stability argument, and competition-instability argument, we think that competition-stability theory holds in the Chinese banking industry. This argument is also supported by Tan and Anchor (2017). In order to proceed to the model evaluation, we need to check whether the hypothesis holds according to our data. Table 1 presents the results regarding the impact of loan-based market power on LLRs over total loans ratio. The results indicate that weaker market power (higher levels of competition) leads to lower LLRs over total loans ratios. In other words, our results are in accordance with our expectation.

**Table 1:** results regarding the impact of loan based market power on LLRs over total loans ratio

LLRs over total loan ratio	Coeffient	Standard error	t	P > t
Loan based market power	0.41	0.03	4.89	0.003
Constant	0.35	0.009	9.75	0.001
Number of observations	430			
F-statistic	585.93			
Prob > F	0.0000			
R square	0.9983			
Adjusted R square	0.9953			

Before presenting the results of efficiency scores considering the dual role played by the LLRs, as well as making comparisons between the scenarios when LLRs are treated as good/desirable outputs, bad/undesirable output and when LLRs are excluded from the model, we summarize the number of bank-year observations for all the three scenarios. The results are presented in Table 2. The table shows that the total number of bank-year observations (when LLRs are excluded from the banking production process) is 430, while there are more bank-year observations that treat LLRs as good/desirable outputs than the ones of bad/undesirable outputs. Therefore, we could conclude that the number of bank-year observations of Chinese banks keeping less than 1% ratio of LLRs over total loans is lower than that of more than 1% ratio. There is a regulatory requirement in the Chinese banking industry to keep the ratio between loan loss reserves and total loans more than 1%, therefore, it is totally understandable that very few observations have the ratio less than 1%, in other words, under the requirement of banking regulatory authority, very few banks did not abide by the regulation. In summary, low number of observations with less than 1% for the ratio between LLRs and total loans is within the expectation in the Chinese banking industry and our findings can be generalized to the whole Chinese banking industry. Looking at this table in further detail, we can observe that when LLRs are treated as good/desirable outputs in the production process, the inefficiency level is the highest, followed by the scenario when LLRs are treated as bad/undesirable output, we find that when LLRs are excluded from the production process, the level of inefficiency is the lowest. In addition, it is observed that when LLRs are treated as good/desirable outputs, there is a biggest difference in the level of inefficiency. The level of difference in inefficiency achieved when LLRs are treated as bad/undesirable outputs is smaller than that the one when LLRs are excluded from the production process.

**Table 2:** descriptive statistics of the inefficiency scores when LLRs are treated as desirable outputs, undesirable output or loan loss provisions are excluded

Inefficiency/statistics	Observations	Mean	Standard deviation	Minimum	Maximum
LLRs are treated as desirable outputs	403	13.03706578	16.68794921	0	66.38484
LLRs are treated as undesirable outputs	27	3.243964444	0.917662641	0.89069	9.67313
LLRs ae excluded from the banking production process	430	1.579162233	4.656852897	0	88.1278

**Fig. 2:**
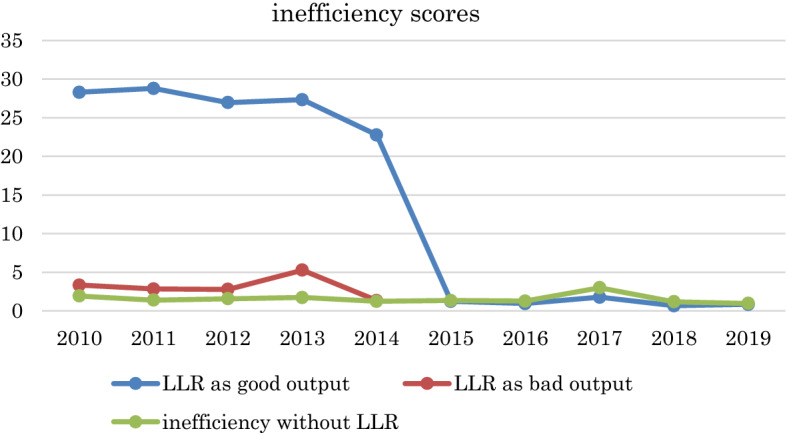
Inefficiency scores when loan loss provisions are treated as good outputs, bad outputs and without consideration of loan loss provisions

We further look at the efficiency scores under these three different scenarios (when LLRs are treated as good/desirable outputs, LLRs are treated as bad/undesirable outputs and LLRs are excluded from the banking production process) on an annual basis. The findings are reported in Fig. 2. It is noticed that when the LLPs are treated as good/desirable outputs, the inefficiency level is the highest for the first four years of the examined period (2010–2014), while the difference in the inefficiency level between the scenarios when LLRs are treaded as good output and LLRs are excluded from the production process is very small for the rest of the period with lowest level of inefficiency observed when LLRs are treated as bad/undesirable outputs. In particular, we observe that there is a substantial decrease in the level of inefficiency after 2014, this is mainly attributed to the fact that over the period 2015–2019, all the Chinese commercial banks in the sample keep the LLRs ratio above 1%, in other words, there are no undesirable outputs generated over this period, the only desirable outputs generated leads to a significant increase in the level of efficiency compared to the previous years when there is a mixture of desirable and undesirable outputs generated. From the perspective of the Chinese banking context, a substantial improvement in the efficiency level after 2014 can be possibly attributed to the fact that China had been finalizing the interest rate literalization, the resulted increase in the competition condition provides more incentives to the bank managers to improve performance.

Our findings are in contrast with Tan and Tsioans (2020) who report that there is a high level of internal efficiency (economic efficiency as measured by the output income and stability efficiency as measured by loan loss provisions), the different results obtained are mainly attributed by the fact that different methods were adopted for the efficiency analysis and different risk indicators were used.

We have a deeper investigation in order to provide important policy implications in relation to the level of LLRs held by various types of banks according to the level of size, bank liquidity and level of equity capital,^5^ the results of which are shown in Table 3. We find that when LLRs are treated as good/desirable outputs, big banks have higher levels of inefficiency, while smaller banks are found to have higher levels of inefficiency when LLRs are excluded from the production process. The findings show that big banks undertake more than prudential behaviour in the banking production process by always keeping the LLRs to total loans ratio of 1%. This finding is in accordance with Tan and Floros (2013a). It is shown that no matter in what way that LLRs are treated in the production process (i.e. as good/desirable outputs, bad/undesirable outputs or excluded from the production process), more liquid banks have higher levels of inefficiency. Our results are in contrast with Sakouvogui (2020) in terms of the US banking industry. The main difference is mainly attributed to the fact that different banking systems are investigated and different efficiency estimation methods are adopted. Our finding can be explained by the fact that Chinese banks with higher level of liquidity reduces the borrowing cost and further lead to an increase in the level of income. Finally, it is found that lower capitalized banks are less efficient when LLRs are treated as good/desirable outputs and excluded from the production process. Our finding is in line with Bitar et al. (2018) with regards to the banks of OECD countries. Our results show that higher capitalized banks always keep the LLRs to total loans ratio of more than 1% every year.

**Table 3:** Inefficiency scores according to size, liquidity and capital

	Number of banks	Inefficiency with LLRs as good/desirable output	Inefficiency with LLRs as bad/undesirable output	Inefficiency without consideration of LLRs in the production process
Bank size				
Big banks	4	13.22	n/a	1.584
Small and medium banks	39	13.13	3.3	1.8
Bank liquidity				
banks with lower level of liquidity	20	12.92	2.68	1.475
banks with higher level of liquidity	23	14.18	3.91	1.88
Bank capital				
banks with larger equity capital	8	9.85	n/a	0.213
banks with lower equity capital	35	14.71	3.13	1.836

## Conclusions and discussions

The banking industry has been and will be always the most important economic sector, considering its vital function of channelling funds from the party with extra money to another party with investment opportunities. The transfer of money will improve the economic efficiency as well as contribute to the economic growth. Unlike other developed countries, the reliance of the Chinese economy on the banking sector is even stronger because of its less developed stock market, insurance industry and the trust industry. The Chinese government has consistently taken efforts to reform the banking industry with the purpose of improving its performance and competitiveness. Nowadays, all the countries across the globe have been increasingly aware that sustainability or sustainable development is even more important than the enhancement of profitability in the banking sector.

One aspect of sustainability is stability, lower risk would be helpful to improve bank stability and the enhancement in bank stability will further contribute to sustainable development. Not only is the stability issue of concern to the government and bank regulatory authority, but academic researchers have also engaged in comprehensive and thorough research into the issue of stability in the banking industry. The topics of research in this area mainly focus on but are not limited to: bank performance and bank stability, bank stability and corporate governance; bank stability and ownership, as well as bank stability and the real economy.

In particular, the investigation of bank stability and bank performance attracts the greatest attention. One group of researchers focuses on a two-phase analysis on this topic, with the first phase using the accounting indicators reflecting profitability and then the second phase using regression analysis through different econometric methods, such as fixed effect, random effect and generalised method of moments to examine the impact or interrelationships between bank stability and bank profitability. Within this group, some studies evaluate efficiencies using either a parametric or non-parametric method (Abdullah & Tan, 2017; Tan, 2018; Nazir et al., 2020; Fukuyama & Tan, 2021b). The second group of researchers, instead of engaging in a two-phase analysis, focus on one phase analysis incorporating risk indicators in the efficiency analysis (Barros et al., 2012; Delis et al., 2017; Tan et al., 2021; Tan & Tsionas, 2020). In other words, for this group, the researchers apply the operational research methods in solving the issues in the finance sector. A number of advanced operational research methods have been applied to the banking sector incorporating the risk indicator. The efficiency analysis includes Bayesian stochastic frontier analysis with undesirable outputs as well as NDEA with undesirable outputs, among others.

When using advanced operational methods incorporating risk indicators to evaluate bank efficiency, the empirical studies mainly focus on employing the “real” risk indicator, namely non-performing loans, while very few studies make attempts to think about the risk from the perspective related to banks' ability to withstand the potential negative shocks. One of these indicators is LLPs. The general provision criteria in the Chinese banking sector is made to improve banks’ ability to absorb negative losses. The criteria formulate that banks should keep at least 1% of total loans as the LLRs by the end of the year for stability purpose. This can be interpreted as if the LLRs to total loans ratio is more than 1%. It reflects that bank risk is low, otherwise if the ratio is less than 1%, it indicates higher risk.

The current study makes significant contributions to the existing studies on operational research in banking efficiency in the following ways: (1) we are the first to make the attempt to propose the dual role played by the LLRs in the banking production process. We argue that LLRs can be regarded as a good/desirable output if the LLRs over total loan ratio is more than 1%, while the LLRs will be treated as an undesirable/bad output if the ratio is less than 1%. The consideration of this dual role opens a new area of operational research in bank efficiency incorporating risk factors; (2) we are the pioneers in considering the costly disposability between loan based market power and undesirable LLRs; 3) we compare the efficiency scores among three different scenarios: a. inefficiency score when LLRs are treated as a good/desirable output; b inefficiency score when LLRs are regarded as an undesirable output; c. inefficiency score when LLRs are excluded from the production process; 4) we compare the level of efficiency under the above three scenarios according to the level of bank size, bank liquidity and bank equity capital.

The results show that the banks with the ratio between LLRs and total loans less than 1% have higher level of efficiency compared to the ones holding the ratio greater than 1%. We compare our results with the one investigating the impact of loan loss reserves on bank efficiency in the emerging Asian countries under a maximum likelihood function by Sun and Chang (2011). Although Sun and Chang (2011) did not have a threshold of 1% for the loan loss reserves, their results show that higher levels of loan loss reserves in total loans result in lower levels of efficiency, this is in line with our results. The results show that when excluding LLRs in the production process, the efficiency scores are significantly inflated. Using the parametric stochastic frontier analysis, Delis et al. (2017) investigate the efficiency of US banks and in particular, they look at the variance of efficiency affected by the role played by the risk factors in the banking production process. Their finding shows that incorporating risk in the stochastic frontier analysis leads to a lower efficiency. Therefore, our result is in accordance with this finding. We find that small and medium sized banks are more efficient than their big counterparts, however, the results show that big banks hold more than enough amounts of LLRs than the one required by the regulatory authority. Our finding is in contrast with Zhu et al. (2020), which reports that large Chinese commercial banks performance better than small and medium sized banks. We explain the difference between the findings of these two studies by the fact that we use more banks in the sample and we consider the inputs and outputs more carefully and comprehensively in the production process. When LLRs are excluded from the production process, it shows that big banks perform better than small and medium sized banks. Our findings show that less liquid banks perform better than the ones with higher levels of liquidity no matter in which way LLRs are treated. This result accords with Tan and Floros (2013b), which reports that under a three-stage least square estimation, bank liquidity is negatively related to bank efficiency in China. Finally, we find that lower capitalized banks, compared to the ones with high levels of capitalization, are less efficient. however, it shows that higher capitalized banks consistently keep more than 1% LLRs out of total loans. Our result is in accordance with Tecles and Tabak (2010), which reports that there is a positive impact of capitalization on bank efficiency in Brazil. The same results obtained between these two studies are derived from two different techniques. More specifically, we reply on the non-parametric data envelopment analysis, while Tecles and Tabak (2010) uses the Bayesian stochastic frontier approach.

Relevant policies can be generated from our findings as below: 1) prioritized attention should by paid to the improvement in efficiency of large banks; 2) stricter capital regulation can be formulated requiring banks to be more capitalized; 3) the banks are recommended to engage in a relatively longer terms of credit allocation, this seems to have a positive impact on efficiency; 4) LLRs are used to absorb the unexpected external shocks, part of which can be alleviated or avoided by putting more effort in the process of risk monitoring and management, therefore, it is recommended that more efforts and emphasis should be given to closely monitoring and managing the risk of business firms, this leaves the banks with the possibility of setting aside a lower proportions of LLRs, which further improve the efficiency level; 5) from the government perspective, more funding should be provided to enhance the practice of financial innovation, the main focus of which is to further reduce the risk that can be more accurately detected by technological innovations and the requirement of holding 1% LLRs out of total loans can be relax thereafter.

In the future, the research could look at the 1% LLRs over total loans ratio in a more careful and thorough manner. For instance, for the bank-year observations with LLRs over total loans of more than 1%, this can be divided into both desirable/good outputs as well as undesirable/bad outputs. 1% amount of LLRs will be regarded as a good/desirable output, any additional percentage amount of LLRs will be regarded as bad/undesirable output. For the bank-year observations with LLRs over total loans of less than 1%, the percentage amount of LLRs can be treated as good/desirable outputs, while the amount of LLRs needed to be held to reach the 1% threshold can be regarded as undesirable/bad outputs. Through this different analysis, the researcher can see whether similar results will be obtained. In addition, future research can consider the non-performing loans and LLRs simultaneously in the production process, the undesirability of the former together with the dual role of the latter would provide more robust findings about the efficiency in the Chinese banking industry. Furthermore, further extensions can be made to estimate different types of efficiencies from different banking perspective, for instance, the overall (in)efficiency in the current study can be further decomposed into economic (in)efficiency and stability (in)efficiency as reflected by the outputs income and LLRs, respectively. Finally, robustness of the results can be obtained in terms of the impact of bank size, liquidity and capitalization on bank efficiency through a second-stage regression analysis.

## Appendix 1

Standard NSBI measure that does not incorprate loan loss provisions

$$ \begin{gathered} {\text{std - }}NSBI\left( {{\mathbf{x}},\;{\mathbf{y}};\;{\mathbf{g}}} \right) \hfill \\ = \max \left\{ {\frac{1}{2}\left( \begin{gathered} \frac{1}{N}\sum\limits_{n = 1}^{N} {\frac{{s_{n}^{x} }}{{g_{n}^{x} }}} \hfill \\ + \frac{1}{M}\sum\limits_{m = 1}^{M} {\frac{{s_{m}^{y} }}{{g_{m}^{y} }}} \hfill \\ \end{gathered} \right)\left| \begin{gathered} \;{\mathbf{x}} - {\mathbf{s}}^{x} \ge \sum\nolimits_{j = 1}^{J} {{\mathbf{x}}_{j} \lambda_{j}^{1} } ,\;\;{\mathbf{z}}^{(1,2)} \le \sum\nolimits_{j = 1}^{J} {{\mathbf{z}}_{j}^{(1,2)} \lambda_{j}^{1} } ,\;\;\sum\nolimits_{j = 1}^{J} {\lambda_{j}^{1} } = 1,\;\;\lambda_{j}^{1} \ge 0\;\;\forall j,\; \hfill \\ \;{\mathbf{z}}^{(1,2)} \ge \sum\nolimits_{j = 1}^{J} {{\mathbf{z}}^{(1,2)} }_{j} \lambda_{j}^{2} ,\;\;{\mathbf{z}}^{(2,3)} \le \sum\nolimits_{j = 1}^{J} {{\mathbf{z}}_{j}^{(2,3)} \lambda_{j}^{2} } ,\;\;\sum\nolimits_{j = 1}^{J} {\lambda_{j}^{2} } = 1,\;\;\lambda_{j}^{2} \ge 0\;\;\forall j, \hfill \\ \;{\mathbf{z}}^{(2,3)} \ge \sum\nolimits_{j = 1}^{J} {{\mathbf{z}}_{j}^{(2,3)} }_{j} \lambda_{j}^{3 - 1} ,\;\;{\mathbf{y}} + {\mathbf{s}}^{y} \le \sum\nolimits_{j = 1}^{J} {{\mathbf{y}}_{j} \lambda_{j}^{3} } ,\;\;\sum\nolimits_{j = 1}^{J} {\lambda_{j}^{3} } = 1,\;\;\lambda_{j}^{3} \ge 0\;\;\forall j \hfill \\ \;{\mathbf{z}}^{(1,2)} \ge {\mathbf{0}},\;\;{\mathbf{z}}^{(2,3)} \ge {\mathbf{0}} \hfill \\ \end{gathered} \right.} \right\} \hfill \\ \end{gathered} $$

**Table A1:** Inputs and Outputs descriptive statistics

Year/statistics	Observations	Mean	Standard deviation	Minimum	Maximum
*Initial inputs*					
Personnel expenses	430	8980.477647	21,436.69545	24.9	116,313
Equity capital	430	141,250.5562	356,240.4539	1301.2	2,365,354
Fixed assets	430	11,350.53994	28,953.58628	2.17	155,480
*Intermediate outputs (deposit generation stage)*					
deposits	430	1,700,931.76	4,005,167.823	1475.1	24,341,438
Deposits-based market power	430	0.023255814	0.053701211	2.99851E-05	0.274974065
Intermdiate outputs (loan generation stage)					
loans	430	1,046,882.725	2,591,645.822	660.1	15,935,564
Loans-based market power	430	0.023255814	0.055597814	2.27098E-05	0.255923322
*Final outputs (income generation stge)*					
Desirable/good outputs					
Net income	430	20,637.82721	53,249.10838	− 231.2	297,325
Dual output					
Loan loss reserves	430	27,660.6711	69,949.67978	11.4	505,724

● The unit of the variables in the table is 100 million RMB

## Footnotes

1 It had been merged with the China Insurance Regulatory Commission since the 13th March 2018.

2 The Russell directional distance function is equivalent to a slack-based measure of inefficiency (Fukuyama & Weber, 2009; Fukuyama & Weber, 2010).

3 Each year banks set aside a certain amount of money available in the form of LLPs, while the accumulation of LLPs over certain periods makes up the loan loss reserves (Monokroussos et al. 2017).

4 The difference between LLRs and LLPs lies in the fact that the former is the accumulation of the latter over the period. See also Footnote 2.

5 We divide the banks into two groups according to size, bank liquidity and equity capital. In the sample, we have 4 state-owned commercial banks, they are defined as big banks, the rest of the banks are small and medium sized banks, liquidity is measured by loans-to-deposits ratio (Van de End, 2016). The banks with an average liquidity ratio over the period higher than the average value of the sample are defined as banks with lower liquidity, other banks are defined as the ones with higher liquidity, the banks with an average equity capital over the period higher than the average value of the sample are defined as banks with higher equity capital, other banks are defined as lower capitalized banks.

## References

[CR1] Abdullah N, Tan Y (2017). Profitability of commercial banks revisited: New evidence from oil and non-oil exporting countries in the MENA region. Investment Management and Financial Innovations.

[CR2] Akther S, Fukuyama H, Weber W (2013). Estimating two-stage network slacks-based inefficiency: An application to Bangledesh banking. Omega.

[CR3] Albaity M, Mallek RS, Noman AHM (2019). Competition and bank stability in the MENA region: The moderating effect of Islamic versus conventional banks. Emerging Markets Review.

[CR4] An Q, Chen H, Wu J, Liang L (2015). Measuring slacks-based efficiency for commercial banks in China by using a two-stage DEA model with undesirable output. Annals of Operations Research.

[CR5] Antunes J, Hadi-Vencheh A, Jamshidi A, Tan Y, Wanke P (2021). Bank efficiency estimation in China: DEA-RENNA approach. Annals of Operations Research.

[CR6] Assaf AG, Matousek R, Tsionas EG (2013). Turkish banking efficiency: Bayesian estimation with undesirable outputs. Journal of Banking and Finance.

[CR7] Bai G, Elyasiani E (2013). Bank stability and managerial compensation. Journal of Banking and Finance.

[CR8] Barros CP, Managi S, Matousek R (2012). The technical efficiency of the Japanese Banks: Non-radial directional performance measruement with undesirable output. Omega.

[CR9] Beck T, De Jonghe O, Schephens G (2013). Bank competition and stability: Cross-country heterogeneity. Journal of Financial Intermediation.

[CR10] Bitar M, Pukthuanthong K, Walker T (2018). The effect of capital ratios on the risk, efficiency and profitability of banks: Evidence from OECD countries. Journal of International Financial Markets, Institutions and Money.

[CR11] Bitar M, Saad W, Benlemlih M (2016). Bank risk and performance in the MENA region: The importance of capital requirement. Economic Systems.

[CR100] Boyd JH, De Nicole G (2005). The theory of bank risk taking and competition revisited. Journal of Finance.

[CR12] Chalermchatvichien P, Seksak J, Pronsit J, Manohar S (2014). The effect of bank ownership concentration on capital adequacy, liquidity, and capital stability (Basel II and Basel III*)*. Journal of Financial Services Research.

[CR13] Chang TC, Chiu YH (2006). Affecting factors on risk-adjusted efficiency in Taiwan’s banking industry. Contemporary Economic Policy.

[CR14] Chao C, Yu M, Wu H (2015). An application of the dynamic network DEA model: The case of banks in Taiwan. Emerging Markets Finance and Trade.

[CR15] Chiu Y, Chen Y, Bai X (2011). Efficiency and risk in Taiwan banking: SBM super-DEA estimation. Applied Economics.

[CR16] Dakpo KH, Jeanneaux P, Latruffe L (2016). Modelling pollution-generating technologies in performance benchmarking: Recent developments, limits and future prospects in the nonparametric framework. European Journal of Operational Research.

[CR17] Delis M, Iosifidi M, Tsionas MG (2017). Endogenous bank risk and efficiency. European Journal of Operational Research.

[CR18] Deng S, Elyasiani E, Jia J (2013). Institutional ownership, diversification, and riskiness of bank holding companies. The Financial Review.

[CR19] Drakos AA, Kouretas GP, Tsoumas C (2016). Ownership, interest rates and bank risk-taking in central and Eastern European Countries. International Review of Financial Analysis.

[CR20] Fang J, Lau CKM, Lu Z, Tan Y, Zhang H (2019). Bank performance in China: A Perspective from Bank efficiency, risk-taking and market competition. Pacific-Basin Finance Journal.

[CR21] Färe R, Grosskopf S (2009). A comment on weak disposability in nonparametric analysis. American Journal of Agriculture Economics.

[CR22] Fiordelisi F, Marques-Ibanez D, Molyneux P (2011). Efficiency and risk in European banking. Journal of Banking and Finance.

[CR23] Førsund FR (2009). Good modelling of bad outputs: Pollution and multiple-output production. International Review of Environmental and Resource Economics.

[CR24] Førsund FR (2018). Multi-equation modelling of desirable and undesirable outputs satisfying the materials balance. Empirical Economics.

[CR25] Fu X, Lin Y, Molyneux P (2014). Bank competition and financial stability in Asia Pacific. Journal of Banking and Finance.

[CR26] Fujii H, Managi S, Matousek R (2014). Indian bank efficiency and productivity changes with undesirable outputs: A disaggregated approach. Journal of Banking and Finance.

[CR27] Fukuyama, H., & Tan, Y. (2020). Deconstructing three-stage overall efficiency into input, output and stability efficiency components with consideration of market power and loan loss provision: An application to Chinese banks. *International Journal of Finance and Economics*. 10.1002/ijfe.2185

[CR28] Fukuyama H, Matousek R (2011). Efficiency of Turkish banking: Two-stage system variable returns to scale model. Journal of International Financial Markets, Institutions and Money.

[CR29] Fukuyama H, Matousek R (2017). Modelling bank performance: A network DEA approach. European Journal of Operational Research.

[CR30] Fukuyama H, Tan Y (2021). Implementing strategic disposability for performance evaluation: Innovation, stability, profitability and corporate social responsibility in Chinese banking. European Journal of Operational Research.

[CR31] Fukuyama H, Tan Y (2021). Corporate social behaviour: Is it good for efficiency in the Chinese banking industry?. Annals of Operations Research.

[CR32] Fukuyama H, Weber WL (2009). A directional slacks-based measure of technical inefficiency. Socio-Economic Planning Sciences.

[CR33] Fukuyama H, Weber WL (2010). A slacks-based inefficiency measure for a two-stage system with bad outputs. Omega.

[CR35] Gaganis C, Lozano-Vivas A, Papadimitri P, Pasiouras F (2020). Macroprudential policies, corporate governance and bank risk: Cross-country evidence. Journal of Economic Behaviour and Organization.

[CR36] Konara P, Tan Y, Johnes J (2019). FDI and heterogeneity in bank efficiency: Evidence from emerging markets. Research in International Business and Finance.

[CR37] Laeven L, Levine R (2009). Bank governance, regulation and risk taking. Journal of Financial Economics.

[CR38] Lee CC, Hsieh MF (2014). Bank reforms, foreign ownership, and financial stability. Journal of International Money, and Finance.

[CR39] Liu J, Tone K (2008). A multistage method to measure efficiency and its application to Japanese banking industry. Socio-Economic Planning Science.

[CR40] Lozano S (2016). Slacks-based inefficiency approach for general networks with bad outputs: An application to the banking sector. Omega.

[CR41] Matthews K (2013). Risk management and managerial efficiency in Chinese banks: A network DEA framework. Omega.

[CR42] Monokroussos, P., Thomakos, D., Alexopoulos, T. A., Tsioli, E. L. (2017). The Determinants of Loan Loss Provisions: An Analysis of the Greek Banking System in Light of the Sovereign Debt Crisis. In P. Monokroussos., & C. Gortsos (Ed), Non-Performing Loans and Resolving Private Sector Insolvency. Cham: Palgrave Macmillan. pp 181–225.

[CR43] Murty S, Russell RR, Levkoff SB (2012). On modeling pollution-generating technologies. Journal of Environmental Economics and Management.

[CR44] Nazir MI, Tan Y, Nazir MR (2020). Intellectual capital performance in the financial sector: Evidence from China, Hong Kong, and Taiwan. International Journal of Finance and Economics.

[CR45] Nguyen M, Skully M, Perera S (2012). Market power, revenue diversification and bank stability: Evidence from selected south asian countries. Journal of International Financial Markets, Institutions and Money.

[CR47] Pak O (2019). The impact of state ownership and business models on bank stability: Empirical evidence from the Eurasian Economic Union. The Quarterly Review of Economics and Finance.

[CR48] Puri J, Yadav SP (2014). A fuzzy DEA model with undesirable fuzzy outputs and its application to the banking sector in India. Expert Systems with Applications.

[CR49] Ray, S. C., & Mukherjee, K. (2007). Efficiency in Managing the Environment and the Opportunity Cost of Pollution Abatement. Working paper, University of Connecticut. Available on the internet at https://media.economics.uconn.edu/working/2007-09.pdf (accessed 09.03.2021)

[CR50] Ray SC, Mukherjee K, Venkatesh A (2018). Nonparametric measures of efficiency in the presence of undesirable outputs: A by-production approach. Empirical Economics.

[CR51] Sakouvogui K (2020). Impact of liquidity and solvency risk factors on variations in efficiency of US banks. Managerial Finance.

[CR52] Srivastav A, Hagendorff J (2015). Corporate governance and bank risk-taking. Corporate Governance: An International Review.

[CR53] Sturn JE, Williams B (2004). Foreign bank entry, deregulation and bank efficiency: Lessons from the Australian experience. Journal of Banking and Finance.

[CR54] Sun L, Chang TP (2011). A comprehensive analysis of the effects of risk measures on bank efficiency: Evidence from emerging Asian countries. Journal of Banking and Finance.

[CR55] Tan Y (2014). Performance, risk and competition in the Chinese banking industry.

[CR56] Tan Y (2016). Efficiency and competition in Chinese banking.

[CR57] Tan Y (2016). The impacts of risk and competition on bank profitability in China. Journal of International Financial Markets, Institutions and Money.

[CR58] Tan Y (2018). The Impacts of Competition and Risk on Profitability in Chinese Banking: Evidence from Boone Indicator and Stability Inefficiency. Annals of Economics and Finance.

[CR59] Tan Y, Anchor J (2017). Does competition only impact on insolvency risk? New evidence from the Chinese banking industry. International Journal of Managerial Finance.

[CR60] Tan Y, Floros C (2013). Market power, stability and performance in the Chinese banking industry. Economic Issues.

[CR61] Tan Y, Floros C (2013). Risk, capital and efficiency in Chinese banking. Journal of International Financial Markets, Institutions and Money.

[CR62] Tan Y, Floros C (2018). Risk, competition and efficiency in banking: Evidence from China. Global Finance Journal.

[CR63] Tan Y, Floros C (2019). Risk, competition and cost efficiency in the Chinese banking industry. International Journal of Banking, Accounting and Finance.

[CR64] Tan Y, Floros C, Anchor J (2017). The profitability of Chinese banks: Impacts of risk, competition and efficiency. Review of Accounting and Finance.

[CR65] Tan Y, Tsionas M (2020). Modelling sustinability efficiency in banking. International Journal of Finance and Economics.

[CR66] Tan Y, Wanke P, Antunes J, Emrouznejad A (2021). Unveiling endogeneity between competition and efficiency in Chinese banks: A two-stage network DEA and regression analysis. Annals of Operations Research.

[CR67] Tecles PL, Tabak BM (2010). Determinants of bank efficiency: The case of Brazil. European Journal of Operational Research.

[CR68] Tsolas IE, Charles V (2015). Incorporating risk into bank efficiency: A satisficing DEA approach to assess the Green banking crisis. Expert Systems with Applications.

[CR69] Van de End JW (2016). A macroprudential approach to address liquidity risk with the loan-to-deposit ratio. The European Journal of Finance.

[CR70] Wang K, Huang W, Wu J, Liu Y (2014). Efficiency measures of the Chinese commercial banking system using an additive two-stage DEA. Omega.

[CR71] Wanke P, Azad MAK, Emrouznejad A, Antunes J (2019). A dynamic network DEA model for accounting and financial indicators: A case of efficiency in MENA banking. International Review of Economics and Finance.

[CR72] Wanke P, Barros CP, Faria JR (2015). Finacial distress drives in Brazilian banks: A dynamic slacks appoach. European Journal of Operational Research.

[CR73] Zha Y, Liang N, Wu M, Bian Y (2016). Efficiency evaluation of banks in China: A dynamic two-stage slacks-based measure approach. Omega.

[CR74] Zhang J, Jiang C, Qu B, Wang P (2013). Market concentration, risk-taking, and bank performance: Evidence from emerging economies. International Review of Financial Analysis.

[CR75] Zhu N, Hougaard JL, Yu Z, Wang B (2020). Ranking Chinese commercial banks based on their expected impact on structural efficiency. Omega.

